# periodicDNA: an R/Bioconductor package to investigate k-mer periodicity in DNA

**DOI:** 10.12688/f1000research.51143.1

**Published:** 2021-02-24

**Authors:** Jacques Serizay, Julie Ahringer

**Affiliations:** 1 The Gurdon Institute and Department of Genetics, University of Cambridge, Cambridge, CB2 1QN, UK

**Keywords:** DNA periodicity, gene regulation, promoter

## Abstract

Periodic occurrences of oligonucleotide sequences can impact the physical properties of DNA. For example, DNA bendability is modulated by 10-bp periodic occurrences of WW (W = A/T) dinucleotides. We present periodicDNA, an R package to identify k-mer periodicity and generate continuous tracks of k-mer periodicity over genomic loci of interest, such as regulatory elements. periodicDNA will facilitate investigation and improve understanding of how periodic DNA sequence features impact function.

## Introduction

Short DNA sequence motifs provide key information for interpreting the instructions in DNA, for example by providing binding sites for proteins or altering the structure of the double-helix. A less studied but important feature of DNA sequence motifs is their periodicity ^1– 4^. Two famous examples are the universal 3bp periodic (RNY) _n_ pattern (R = A or G, Y = C or U, N = any base) in exons ^5^ and 10-bp periodic k-mers in nucleosome positioning (reviewed in 6 and 7). However, despite the wealth of software focusing on motif discovery and analysis, no tool provides an easy way to quantify the periodicity of a given motif, i.e. the extent to which a given motif is repeated at a regular interval in specific sequences. For instance, HeliCis and SpaMo identify conserved distances between two motifs in sequences of interest, but they do not assess larger scale periodic occurrences of motifs ^8, 9^.

Here we present periodicDNA, an R package to investigate k-mer periodicity. periodicDNA provides a framework to quantify the periodicity of any k-mer of interest in DNA sequences. It can identify which periods are statistically enriched in a set of sequences by using a randomized shuffling approach to compute an empirical p-value and can also generate continuous linear tracks of k-mer periodicity strength over genomic loci.

## Methods

### Operation

The two main functions of periodicDNA are getPeriodicity() and getPeriodicityTrack(). The getPeriodicity() function interrogates the extent to which a given k-mer periodically occurs, and at which periods, in one sequence or a set of sequences. It uses a randomized shuffling approach to compute an empirical p-value and identify k-mer periods that are statistically enriched in the sequences of interest. getPeriodicityTrack() generates a linear .bigWig track over genomic loci of interest, representing the periodicity strength of a chosen k-mer at a given period.

periodicDNA is available as an R package on Github. Package dependencies and system requirements are documented here: https://js2264.github.io/periodicDNA/. periodicDNA has been tested using R (version 4.0.2) on Mac OS (versions 10.11 and later) and Ubuntu 18.04.2 machines.

### Implementation

**Figure 1.**
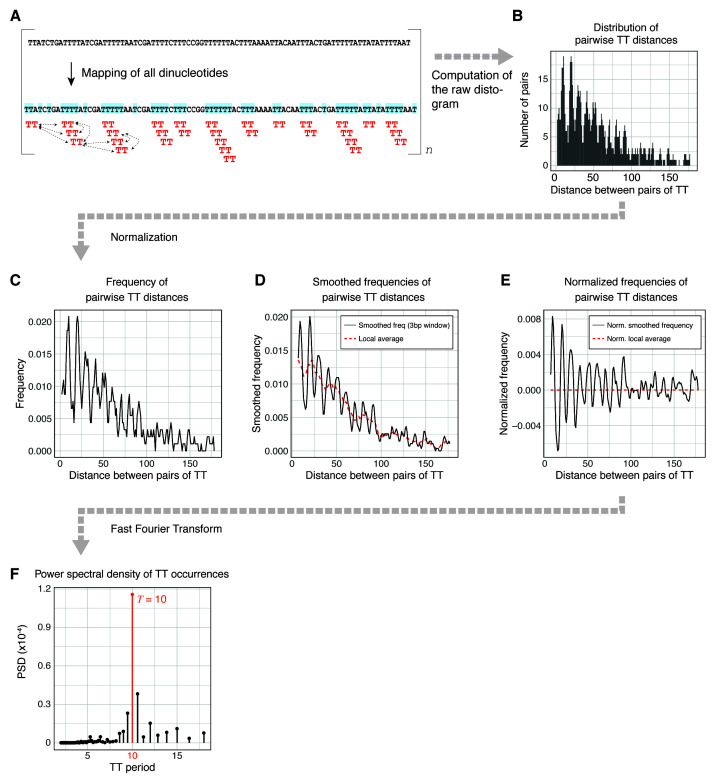
Estimation of k-mer power spectral density with periodicDNA. ( **A**) The k-mer of interest is mapped in the input sequence and all pairwise distances are calculated. ( **B**) The distribution of all resulting pairwise distances (also called the "distogram") is generated. ( **C**) The distogram is transformed into a frequency histogram and ( **D**) smoothed using a moving window of 3 to mask the universal three-base genomic periodicity ^11^. ( **E**) To normalize the frequency for distance decay, the local average (obtained by averaging the frequency with a moving window of 10) is subtracted from the smoothed frequency. ( **F**) Finally, the power spectral density (PSD) is estimated using a Fast Fourier Transform ( **F**). In this example, TT shows strong 10bp periodicity.

Internally, getPeriodicity() and getPeriodicityTrack() functions both compute the power spectral density (PSD) of an input k-mer in input sequence(s) to estimate its average periodicity. The magnitude of the PSD reflects the strength of the k-mer signal at periodic positions ^10^. To compute the power spectral density (PSD) of a k-mer of interest in one or several sequences, the following steps are executed ( Figure 1):

1. The k-mer occurrences are mapped and their pairwise distances are calculated ( Figure 1A).
2. The distribution of all the resulting pairwise distances (also called "distogram") is generated ( Figure 1B).
3. The distogram is transformed into a frequency histogram ( Figure 1C) and smoothed using a moving window of 3 to mask the universal three-base genomic periodicity ^11^ ( Figure 1D). To normalize the frequency for distance decay, the local average (obtained by averaging the frequency with a moving window of 10) is subtracted from the smoothed frequency ( Figure 1E).
4. Finally, the power spectral density (PSD) is estimated using a Fast Fourier Transform ( Figure 1F). The magnitude of the PSD values indicates the contribution of a given period to the overall periodicity of the k-mer of interest. In Figure 1, TT dinucleotides are generally 10bp periodic.

The package relies on BSGenome packages to handle genome assemblies. Genomic loci can be imported from BED files with rtracklayer and are handled in R by the GenomicRanges classes. Biostrings functions are used to map k-mer occurrences in sequences of interest.

## Workflow

### Installation

To install the current release of periodicDNA from Bioconductor, use:


 > if (!requireNamespace("BiocManager", quietly = TRUE))
     install.packages("BiocManager")
 > BiocManager::install("periodicDNA")
 > library("periodicDNA")


Alternatively, the developing branch of periodicDNA can be installed from Github as follows:


 > remotes::install_github("js2264/periodicDNA")
 > library("periodicDNA")


### Quantifying k-mer periodicity over a set of sequences

Using a provided k-mer (e.g. WW, motif argument) and a set of sequences of interest ( seqs argument), getPeriodicity() computes several different metrics:

1. The k-mer power spectral density (PSD) values at different periods obtained by Fourier Transform (following the approach described in the Implementation section). For each individual period *T*, the corresponding PSD _*T*_ indicates the relative contribution of the period to the overall periodicity of the k-mer of interest ^10^.
2. The log2 fold-change, for each individual period *T*, between the observed PSD _*T*_ and the median of the PSD _*T*_ values measured after shuffling n times the input sequences $(l2FC=log2\left(\frac{PSD_{T,observed}}{median(PSD_{T,shuffled})}\right)).$
3. Associated empirical p-values and false discovery rates (FDR) indicating, for each individual period *T*, whether the observed PSD _*T,observed*_ is significantly greater than PSD _*T,shuffled*_ values measured after shuffling n times the input sequences $(p=\frac{\sum_{i=1}^{n}(PSD_{T,shuffled}\geq PSD_{T,observed})+1}{n+1},$ ^12^). Note that empirical p-values are only an estimation of the real p-value. Notably, small p-values are systematically over-estimated as their lower bound is 1/( *n* + 1).

These metrics are accessible in the periodicityMetrics table obtained when running getPeriodicity(). For each Frequency (or Period) analysed by Fourier Transform, the resulting PSD value, a log2 fold-change, its associated p-value as well as its false-discovery rate (FDR) are returned (see tables in the examples below).

**Figure 2.**
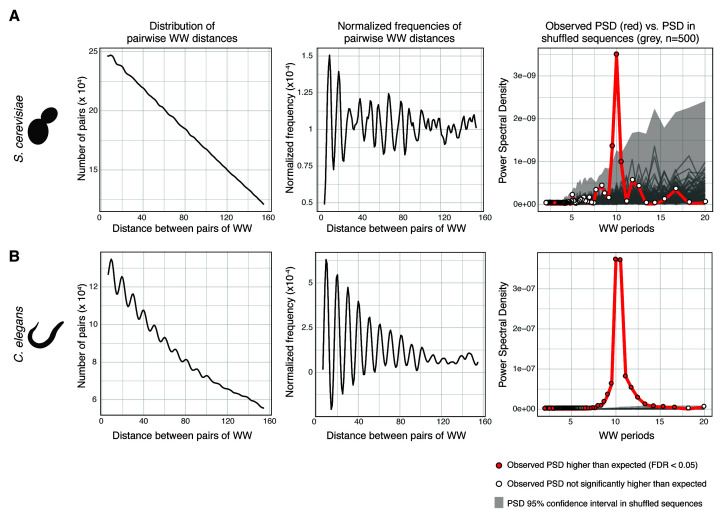
Output of the plotPeriodicityResults() function run on getPeriodicity() results. To identify periodicity of WW dinucleotides, getPeriodicity() was run on ( **A**) a set of 300-bp long sequences centered at 6,533 *S. cerevisiae* TSSs and ( **B**) a set of 300-bp long sequences centered at 2,295 ubiquitous *C. elegans* TSSs ^14^. The plotPeriodicityResults() function was run on the getPeriodicity() results to generate three plots as shown. Left, frequency histogram of distribution of pairwise WW distances; middle, normalised frequency histogram of distribution of pairwise WW distances; right, power spectral densities (PSDs) of a set of experimental sequences (red) and 500 iterations of shuffled sequences (grey). Grey ribbon represents the 95% confidence interval of the PSD values obtained after sequence shuffling. Red-filled dots represent PSD values in experimental sequences statistically higher than those from shuffled sequences (FDR < 0.05).

We ran getPeriodicity() on a set of 6,533 300-bp long sequences centered at all *S. cerevisiae* TSSs, to investigate WW periodicity, comparing to 500 shufflings as default. Using 12 cores in parallel, this function took approximately 15 minutes to run. The results were then plotted using plotPeriodicityResults() ( Figure 2A), demonstrating the known underlying 10-bp WW periodicity present at promoter sequences in the yeast genome ^13^.


 > # --------- Get the sequences of S. cerevisiae TSSs
 > library(TxDb.Scerevisiae.UCSC.sacCer3.sgdGene)
 > library(GenomicFeatures)
 > library(magrittr)
 > genes <- genes(TxDb.Scerevisiae.UCSC.sacCer3.sgdGene)
 > sacCer3_TSSs <- genes %>%
     resize(fix = 'center', 300) %>% 
     '['(. %within% GRanges(seqinfo(genes)))
 > # --------- Run getPeriodicity()
 > sacCer3_results <- getPeriodicity(
     sacCer3_TSSs,
     genome = 'sacCer3', 
     motif = 'WW', 
     n_shuffling = 500, 
     cores_shuffling = 12
   )
 > # --------- Plot results with plotPeriodicityResults()
 > plotPeriodicityResults(sacCer3_results, xlim = 150) 
 > # --------- Print the computed periodicity metrics 
 > sacCer3_results$periodicityMetrics

##  |  Freq|  Period|PSD_observed |    l2FC|  pval   |    fdr|
##  |-----:|-------:|:------------|-------:|:--------|------:|
##  | 0.005| 200.000|3.32e-08     | -0.5313|1.00e+00 | 1.0000|
##  | 0.010| 100.000|5.83e-09     | -0.6259|1.00e+00 | 1.0000|
##  | 0.015|  66.667|1.45e-09     | -0.7594|1.00e+00 | 1.0000|
##  | 0.020|  50.000|9.38e-10     |  0.3728|2.55e-01 | 0.9125|
##  | 0.025|  40.000|1.78e-10     | -0.7144|7.49e-01 | 1.0000|
##  | 0.030|  33.333|3.18e-10     |  0.4657|3.25e-01 | 1.0000|
##  | 0.035|  28.571|1.18e-10     | -0.9550|7.31e-01 | 1.0000|
##  | 0.040|  25.000|3.84e-11     | -2.5141|9.28e-01 | 1.0000|
##  | 0.045|  22.222|6.63e-12     | -4.9433|9.88e-01 | 1.0000|
##  | 0.050|  20.000|3.66e-11     | -1.9689|8.70e-01 | 1.0000|
##  | 0.055|  18.182|2.49e-11     | -2.6349|8.94e-01 | 1.0000|
##  | 0.060|  16.667|3.40e-10     |  1.4118|1.50e-01 | 0.7879|
##  | 0.065|  15.385|9.89e-11     | -0.3535|6.01e-01 | 1.0000|
##  | 0.070|  14.286|1.27e-11     | -3.2144|9.42e-01 | 1.0000|
##  | 0.075|  13.333|1.29e-11     | -3.2219|9.52e-01 | 1.0000|
##  | 0.080|  12.500|4.05e-10     |  1.8788|8.38e-02 | 0.5240|
##  | 0.085|  11.765|5.45e-10     |  2.4637|2.59e-02 | 0.2162|
##  | 0.090|  11.111|4.66e-11     | -0.9672|7.21e-01 | 1.0000|
##  | 0.095|  10.526|9.78e-10     |  3.5220|2.00e-03 | 0.0499|
##  | 0.100|  10.000|3.48e-09     |  5.7209|2.00e-03 | 0.0499|
##  | 0.105|   9.524|1.34e-09     |  4.3497|2.00e-03 | 0.0499|
##  | 0.110|   9.091|1.28e-10     |  1.1208|2.22e-01 | 0.8862|
##  | 0.115|   8.696|2.37e-10     |  2.1408|4.19e-02 | 0.3224|
##  | 0.120|   8.333|4.38e-10     |  3.2951|3.99e-03 | 0.0544|
##  | 0.125|   8.000|3.65e-10     |  3.0512|3.99e-03 | 0.0544|
##  | 0.130|   7.692|3.37e-10     |  3.0458|5.99e-03 | 0.0544|
##  | 0.135|   7.407|7.23e-12     | -2.5883|8.96e-01 | 1.0000|
##  | 0.140|   7.143|1.84e-11     | -1.2455|7.11e-01 | 1.0000|
##  | 0.145|   6.897|1.35e-10     |  1.7676|9.38e-02 | 0.5518|
##  | 0.150|   6.667|3.43e-11     | -0.0344|5.05e-01 | 1.0000|
##  ...


Using the same approach, we measured the WW periodicity around ubiquitous TSSs in *C. elegans*, which have been characterized as largely enriched for WW 10-bp periodic sequences ^14^ ( Figure 2B). The 10-bp WW periodicity at ubiquitous *C. elegans* TSSs ** is stronger than at all *S. cerevisiae* TSSs.


 > # ---------  Run getPeriodicity()
 > data(ce11_TSSs)
 > ce11_results <- getPeriodicity(
     ce11_TSSs[['Ubiq.']],
     genome = 'ce11', 
     motif = 'WW', 
     n_shuffling = 500, 
     cores_shuffling = 12
   )
 > # --------- Plot results with plotPeriodicityResults()
 > plotPeriodicityResults(ce11_results, xlim = 150)
 > # --------- Print the computed periodicity metrics 
 > ce11_results$periodicityMetrics

## | Freq|   Period|PSD_observed |    l2FC|  pval   |    fdr|
## |-----:|-------:|:------------|-------:|:--------|------:|
## | 0.005| 200.000|4.59e-08     |  0.0716|1.04e-01 | 0.2414|
## | 0.010| 100.000|8.96e-09     |  0.1241|2.02e-01 | 0.3733|
## | 0.015| 66.667|8.15e-10      | -1.4410|1.00e+00 | 1.0000|
## | 0.020| 50.000|3.07e-09      |  2.2148|2.00e-03 | 0.0080|
## | 0.025| 40.000|4.48e-09      |  3.7005|2.00e-03 | 0.0080|
## | 0.030| 33.333|1.59e-09      |  2.5195|5.99e-03 | 0.0222|
## | 0.035| 28.571|1.05e-09      |  1.8486|7.78e-02 | 0.2162|
## | 0.040| 25.000|2.88e-10      |  0.0116|4.95e-01 | 0.6770|
## | 0.045| 22.222|1.55e-10      | -1.0142|7.29e-01 | 0.8471|
## | 0.050| 20.000|5.22e-09      |  4.2517|2.00e-03 | 0.0080|
## | 0.055| 18.182|2.18e-10      | -0.2869|5.51e-01 | 0.7155|
## | 0.060| 16.667|3.16e-09      |  3.6181|2.00e-03 | 0.0080|
## | 0.065| 15.385|5.03e-09      |  4.4401|2.00e-03 | 0.0080|
## | 0.070| 14.286|6.47e-09      |  4.6155|2.00e-03 | 0.0080|
## | 0.075| 13.333|1.12e-08      |  5.6508|2.00e-03 | 0.0080|
## | 0.080| 12.500|2.79e-08      |  6.9992|2.00e-03 | 0.0080|
## | 0.085| 11.765|5.27e-08      |  8.0068|2.00e-03 | 0.0080|
## | 0.090| 11.111|8.08e-08      |  8.7938|2.00e-03 | 0.0080|
## | 0.095| 10.526|3.71e-07      | 10.9426|2.00e-03 | 0.0080|
## | 0.100| 10.000|3.72e-07      | 11.3303|2.00e-03 | 0.0080|
## | 0.105|  9.524|6.26e-08      |  8.8359|2.00e-03 | 0.0080|
## | 0.110|  9.091|3.56e-08      |  8.3330|2.00e-03 | 0.0080|
## | 0.115|  8.696|1.75e-08      |  7.4185|2.00e-03 | 0.0080|
## | 0.120|  8.333|7.48e-09      |  6.6215|2.00e-03 | 0.0080|
## | 0.125|  8.000|7.21e-09      |  6.4817|2.00e-03 | 0.0080|
## | 0.130|  7.692|1.43e-09      |  4.1824|2.00e-03 | 0.0080|
## | 0.135|  7.407|1.75e-09      |  4.5476|2.00e-03 | 0.0080|
## | 0.140|  7.143|8.75e-10      |  3.5609|2.00e-03 | 0.0080|
## | 0.145|  6.897|1.04e-09      |  3.7575|2.00e-03 | 0.0080|
## | 0.150|  6.667|4.38e-10      |  2.6277|1.40e-02 | 0.0466|
##  ....


### Generating tracks of k-mer periodicity

**Figure 3.**
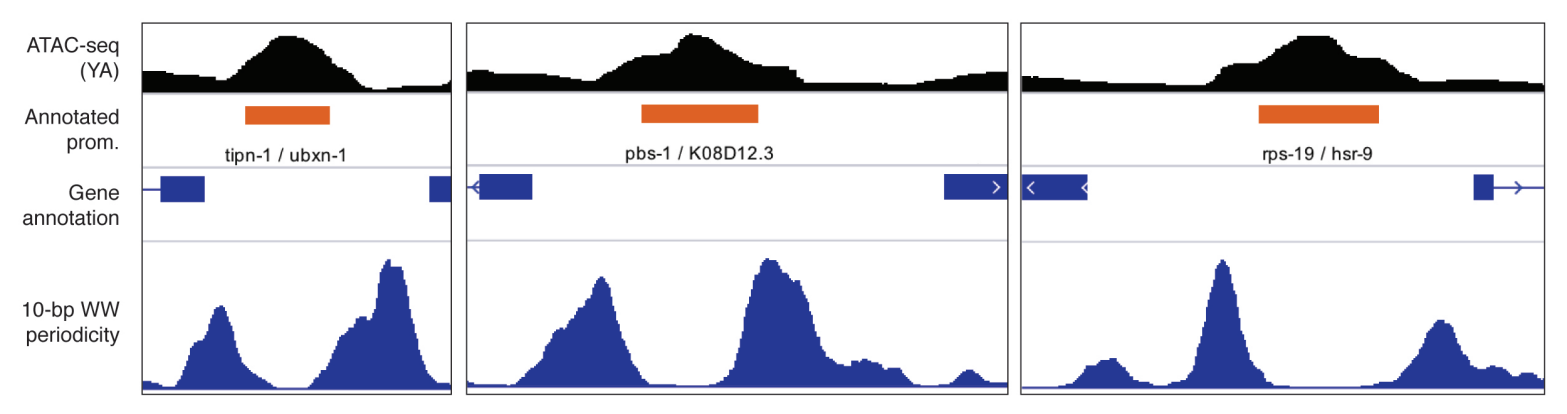
Example of WW dinucleotide 10-bp periodicity track at promoters in *C. elegans*. ATAC-seq signal and 10-bp WW dinucleotide periodicity at three *C. elegans* promoters. 10bp WW dinucleotide periodicity signal was generated at 1 kb centered at annotated *C. elegans* promoters ^14^ using the `generatePeriodicityTrack()` function. The data are vizualised using IGV.

The generatePeriodicityTrack() function calculates the strength of a given k-mer at a particular periodicity across genomic regions of interest, generating a linear genomic track in .bigWig format ( Figure 3). The user specifies a genome and a set of genomic loci, a motif and a period of interest, and a sliding window size ( window_size, 100 bp by default) and step value ( step_size, 2 bp by default). The input genomic loci are split into small sliding windows and for each window, the k-mer periodicity is quantified as described in the Implementation section. The PSD value at the period of interest (e.g. period = 10) is then retrieved and assigned to the center of the corresponding window. Finally, the resulting .bigwig track is smoothed using a rolling window ( smooth_track = 20). generatePeriodicityTrac() should be run in parallel across many cores using the BPPARAM argument from BiocParallel. Using 12 cores, this command takes approximately half a day to produce a periodicity track over ~ 15,000 1-kb-long GRanges with default parameters.

As an example, we generated a WW 10-bp periodicity linear track over annotated promoters in *C. elegans* genome. In the previous section, we have shown that sequences in the vicinity of ubiquitous TSSs were statistically enriched for WW 10-bp periodicity. Here, the .bigwig track highlights the increased WW 10-bp periodicity in the sequences immediately flanking ubiquitous promoters, where the -1 and +1 nucleosomes are positioned ^14^.


 > data(ce11_proms)
 > track <- getPeriodicityTrack(
     genome = 'ce11',
     granges = ce11_proms, 
     motif = 'WW',
     period = 10,
     window_size = 100,
     step_size = 2,
     smooth_track = 20,
     BPPARAM = setUpBPPARAM(12), 
     bw_file = 'WW-10-bp-periodicity_over-proms_ce11.bw'
   )


## Conclusion

periodicDNA is an R package that provides functions to investigate the periodicity of k-mers of interest in DNA sequences. It is primarily designed to analyse individual or sets of sequences (typically few hundred bases long and up to a kilobase) to identify overall periodicity of a chosen k-mer. It can also generate linear .bigwig tracks of k-mer periodicity at a chosen period (e.g. 10-bp WW periodicity), over genomic loci of interest. periodicDNA is well integrated within the Bioconductor environment and can easily fit in standard genomic analysis workflows.

## Data availability

### Underlying data

This project contains underlying data published in Serizay *et al.,* 2020 ^14^. All the data are also available from the original reference.

## Software availability

periodicDNA is released as an R package on Bioconductor: http://www.bioconductor.org/packages/release/bioc/html/periodicDNA.html

Source code available from: https://github.com/js2264/periodicDNA.

Archived source code as at time of publication: https://doi.org/10.5281/zenodo.4533704 ^15^.

License: GPL-3
