# Supplementary material for: periodicDNA: an R/Bioconductor package to investigate k-mer periodicity in DNA
Source: F1000Res. 2021 Feb 24;10:141. [Version 1] doi: 10.12688/f1000research.51143.1 (PMC8063535; doi:10.12688/f1000research.51143.1)
Supplement: Peer review history

# Peer review: Reviewer response for version 1

**DOI:** 10.5256/f1000research.54274.r80117

**Published:** 2021-05-12

**Authors:** Boris Lenhard

**Affiliations:** 1 Institute of Clinical Sciences, Faculty of Medicine, Imperial College London, London, UK

periodicDNA is a R/Bioconductor package for the detection and visualisation of string-based periodic motifs (k-mers) in DNA sequences. While there are several periodic motifs that are known to occur in genomic sentences, the paper - and, for the most part, the tool itself - focuses on the analysis of by far the most important of them, the 10-bp periodicity of WW dinucleotides that is the hallmark of nucleosome positioning signals, present in the majority of RNA polymerase II promoters, in most eukaryotic genomes analysed so far.

 The tool implements the calculation of power spectral density over the distances between the instances of k-mers in a set of sequences by normalising the frequencies of pairwise TT distances against "smoothed" distances. It has only a couple of main functions, one for performing general power spectrum analyses, with table of power spectral density as a result, and one that produces a genome browser track for a selected set of sequences using a predefined period size. The latter is shown to produce a characteristic double pattern at bidirectional promoters in *C. elegans* (Figure 3).

 The tool is simple, clean, and seems to do its job well. I have only a couple of suggestions:

- The smoothed local average (Figure 1D) uses a 10 bp window. While that is a suitable window size for nucleosome positioning signal, it might not work for exploring other, possibly unknown periodic motifs. It should at least be remarked on, and the authors should consider adding functionality to suggest an optimal or at least minimal window size for averaging.
- The stated running times on a 12-core CPU for the presented examples look surprisingly long to me. Could the authors provide more detail - with or without profiling the code - about which computational step is the reason the whole calculation takes this long? 

Are the conclusions about the tool and its performance adequately supported by the findings presented in the article?

Yes

Is the rationale for developing the new software tool clearly explained?

Yes

Is the description of the software tool technically sound?

Yes

Are sufficient details of the code, methods and analysis (if applicable) provided to allow replication of the software development and its use by others?

Partly

Is sufficient information provided to allow interpretation of the expected output datasets and any results generated using the tool?

Yes

Reviewer Expertise:

Gene regulation.

I confirm that I have read this submission and believe that I have an appropriate level of expertise to confirm that it is of an acceptable scientific standard, however I have significant reservations, as outlined above.

# Peer review: Reviewer response for version 1

**DOI:** 10.5256/f1000research.54274.r81592

**Published:** 2021-04-13

**Authors:** Eugene Korotkov

**Affiliations:** 1 Research Center of Biotechnology, Russian Academy of Sciences, Moscow, Russian Federation

The manuscript is aimed at solving an important problem - the study of the structure of promoter sequences. The calculations are performed at a good scientific level, the results are beyond doubt. I have only three, as it seems to me, small comments.

 1. It seems to me that it is not entirely correct to represent the data as a file printout. Probably it would be better to present this data in the form of tables.

 2. Figure 3 evokes some skepticism. It seems to me that it is very difficult to draw a conclusion on 3 promoters. Maybe this is a purely coincidence. I believe that it is necessary to study much more promoters in this way in order to draw any conclusions. Probably more promoter sequences need to be studied.

 3. There are a lot of publications on the search for periodicity in promoter sequences. For example, Kravatskaya GI *et al.* (2011) ^1^ and Nov Klaiman T *et al.* (2009) ^2^

 It seems to me that a little comparative analysis could be done in the discussion.

Are the conclusions about the tool and its performance adequately supported by the findings presented in the article?

Partly

Is the rationale for developing the new software tool clearly explained?

Yes

Is the description of the software tool technically sound?

Yes

Are sufficient details of the code, methods and analysis (if applicable) provided to allow replication of the software development and its use by others?

Yes

Is sufficient information provided to allow interpretation of the expected output datasets and any results generated using the tool?

Yes

Reviewer Expertise:

Bioinformatics

I confirm that I have read this submission and believe that I have an appropriate level of expertise to confirm that it is of an acceptable scientific standard, however I have significant reservations, as outlined above.

## References

[1] : Coexistence of different base periodicities in prokaryotic genomes as related to DNA curvature, supercoiling, and transcription. *Genomics*.2011;98(3) : 10.1016/j.ygeno.2011.06.006 223-31 10.1016/j.ygeno.2011.06.006 21722724

[2] : Upstream curved sequences in E. coli are related to the regulation of transcription initiation. *Comput Biol Chem*.2009;33(4) : 10.1016/j.compbiolchem.2009.06.007 275-82 10.1016/j.compbiolchem.2009.06.007 19646927

# Peer review: Reviewer response for version 1

**DOI:** 10.5256/f1000research.54274.r81589

**Published:** 2021-04-12

**Authors:** Sabarinathan Radhakrishnan

**Affiliations:** 1 National Centre for Biological Sciences, Bangalore, India

The manuscript by Serizay and Ahringer presents an R/Bioconductor package to investigate the k-mer periodicity in DNA. Both the manuscript and the package details are informative and will be useful to the researchers. The following are some minor comments and suggestions:

 a) Regarding the shuffling of input sequences, it is not clear if there is an option to preserve the doublet or triplet nucleotide counts. Is the parameter “order” in getPeriodicity() function meant for that (through ushuffle)? If so, please do mention this in the manuscript and its default value. 

 b) In addition to the shuffling of input sequences, it would also be useful if the function allows for random selection of N number of sequences (with similar GC content) from the genome or an option for the users to define their own set of input random sequences (through DNAstringSet or bed file).

 c) In Figure 1A, under the “Mapping of all dinucleotides” it is not clear why the single occurrence of ‘T’ is also shaded.

Are the conclusions about the tool and its performance adequately supported by the findings presented in the article?

Yes

Is the rationale for developing the new software tool clearly explained?

Yes

Is the description of the software tool technically sound?

Yes

Are sufficient details of the code, methods and analysis (if applicable) provided to allow replication of the software development and its use by others?

Yes

Is sufficient information provided to allow interpretation of the expected output datasets and any results generated using the tool?

Yes

Reviewer Expertise:

Bioinformatics, Cancer genomics, mutational processes and gene regulation.

I confirm that I have read this submission and believe that I have an appropriate level of expertise to confirm that it is of an acceptable scientific standard.

# Peer review: Reviewer response for version 1

**DOI:** 10.5256/f1000research.54274.r81590

**Published:** 2021-03-26

**Authors:** Ilya Ioshikhes

**Affiliations:** 1 University of Ottawa, Ottawa, Canada

The software offered is potentially helpful to the researchers in the area. However the paper (and probably the software itself) should be modified to enable its complete evaluation for indexing.

1. Comparison to other existing state of the art approaches should be added, e.g. to PerPlot and PerScan tools by Mrazek and colleagues ^1^, ID/MPA by Shelenkov *et al*., 2008 ^2^. The progress obtained in the present submission should be clarified.
2. All the examples shown in the paper and related links are focused on TT dinucleotides. It is not clear whether oligo nucleotide combinations involving W, S, R, Y, N, and others from the IUPAC nucleotide codes are supported by the present software version.
3. It is not clear whether there any other restrictions on the software parameters, e.g. oligonucleotide and sequences lengths.
4. The software is composed at R language with key functions provided. To make it really practically helpful, the graphic user interface should be provided, to enable entering oligonucleotides of interest and other parameters from a window or dialogue line, without necessitating the changes in the functions text. As a very minimum, the clear instructions for such changes should be provided.

Are the conclusions about the tool and its performance adequately supported by the findings presented in the article?

Yes

Is the rationale for developing the new software tool clearly explained?

Yes

Is the description of the software tool technically sound?

Yes

Are sufficient details of the code, methods and analysis (if applicable) provided to allow replication of the software development and its use by others?

Yes

Is sufficient information provided to allow interpretation of the expected output datasets and any results generated using the tool?

Yes

Reviewer Expertise:

Bioinformatics, gene regulation, nucleosomes, algorithms

I confirm that I have read this submission and believe that I have an appropriate level of expertise to confirm that it is of an acceptable scientific standard, however I have significant reservations, as outlined above.

## References

[1] : PerPlot & PerScan: tools for analysis of DNA curvature-related periodicity in genomic nucleotide sequences. *Microb Inform Exp*.2011;1(1) : 10.1186/2042-5783-1-13 13 10.1186/2042-5783-1-13 22587738PMC3372288

[2] : Classification analysis of a latent dinucleotide periodicity of plant genomes. *Russian Journal of Genetics*.2008;44(1) : 10.1134/S1022795408010134 101-114 10.1134/S1022795408010134 18409394
